# Rectosigmoid stump washout as an alternative to permanent mucous fistula in patients undergoing subtotal colectomy for ulcerative colitis in emergency settings

**DOI:** 10.1186/1471-2482-12-S1-S31

**Published:** 2012-11-15

**Authors:** Gianluca Pellino, Guido Sciaudone, Giuseppe Candilio, Silvestro Canonico, Francesco Selvaggi

**Affiliations:** 1 Department of Surgery - Unit of General and Geriatric Surgery , School of Medicine, Second University of Naples, Naples, Italy

## Abstract

**Background:**

Restorative proctocolectomy with ileopouch-anal anastomosis (IPAA) is the treatment of choice for intractable or complicated ulcerative colitis(UC). Elderly patients often present with acute colitis requiring emergent subtotal colectomy(SC). Frail patients are at risk of developing septic complications related to the closed rectosigmoidal stump, often requiring formation of a second stoma to be reversed at the time of completion proctectomy. This carries nuisance to such exhausted patients. We propose a simple and inexpensive trick to avoid the need for creating a mucous fistula.

**Methods:**

IPAA was performed as a 3-stage procedure in emergency settings. The rectosigmoidal stump was closed and placed subcutaneously; skin was closed over it. After SC, if patients showed signs of stump-related pelvic sepsis, a lavage of the rectal stump with povidone iodine solution and with saline was carried out as a rescue treatment aiming to avoid the need of opening the rectal stump to drain sepsis.

**Results:**

Thirty-five patients underwent SC for UC between 1987 and 2012. The skin was closed over the closed stump in the 20. Seven patients out of these 20 experienced early stump-related septic complication. In five cases, we were able to avoid opening of the rectal stump, and a second stoma was unnecessary. After opening the closed stump in the remaining ones, a prompt improving of symptoms was observed.

**Conclusions:**

Rectal washout was well tolerated and avoided a second stoma in five out of seven patients, with better quality of life and body perception after IPAA surgery. This is relevant when dealing with geriatric patients, needing to completely recover before undergoing completion proctectomy.

## Background

Restorative proctocolectomy with ileopouch-anal anastomosis (IPAA) is the treatment of choice for intractable or complicated ulcerative colitis(UC). However, when nutritional status and haemoglobin levels are poor or if patient is receiving treatment with steroids pouch fashioning should be delayed and an urgent/emergency subtotal colectomy(SC) with terminal ileostomy and closure of a long rectal (sigmoid) stump is to be preferred [1-3]. This is very common in elderly patients presenting with acute colitis unresponsive to medical treatment. If septic complications related to the stump occur, it is possible to open the tip of the stump, creating a mucous fistula to drain sepsis. However this almost always determines another stoma for the patient to deal with until definitive surgery.

We herein describe a simple method to avoid a second stoma in UC patients undergoing SC with suspicion of early septic complication related to the retained diseased stump.

## Methods

SC was performed via median laparotomy or laparoscopy with a median mini-laparotomy to exteriorize the colon; a terminal ileostomy was fashioned on a site marked preoperatively by scratching the skin with a small needle; in laparoscopy patients, ileostomy was exteriorized through an adequately widened trocar incision. The rectosigmoidal stump was closed with a linear stapler with or without hand-sewn additional stitches, and placed subcutaneously in left iliac fossa or at the end of the median laparotomy incision. In patients undergoing laparoscopy, this was passed through a trocar incision in left iliac fossa. Skin was always closed over it. At the end of the procedure, two silicone drains were placed in the abdominal cavity, positioning one next to the colonic stump.

If signs of local stump-related sepsis occur during postoperative observation (e.g. fever, swelling and/or tenderness in proximity of the skin covering the tip of the stump, high white blood cells (WBC) count), without signs of abdominal involvement (mute/serous-discharging drains, no abdominal defence, no evidence of collections in the pelvis at ultrasonography or pelvic effusion emanating from the stump at radiography with gastrographyn enema), we use the following approach. A soft, silicone three-way catheter, size Ch. 22-24 is introduced through the anus with the patient in Sims’ position and the balloon is insufflated with 30 ml of saline. Then, the patient is placed in mild Trendelemburg position (approximately 30° from the floor) and rectal washout is started through the afferent channel of the catheter with 1000 ml of saline plus 10 ml of Povidone-Iodine solution (PVI, Betadine^®^) (1%): this is completed in approximately 2 hours. Finally, slow washout with abundant saline is carried out. During night-time, the catheter is removed and washout is suspended. The procedure is repeated the next day, but PVI is diluted at 0.1%; saline solution is subsequently infused continuously (approximately 8 hours). The washout can be repeated for additional 12 hours.

During the whole procedure it is mandatory to evaluate general health condition and to assess at least daily the serological status (albumin, total protein count, WBC, haemoglobin). Antibiotic is continued until normalization of WBC and remission of fever. The drains are to be kept in place. Most important, the patient should receive enteral nutrition as soon as possible, with high caloric and protein intake. If there is no response within 12 hours, it is safe to remove cutaneous sutures over the stump, opening it when needed draining sepsis through this mucous fistula. Washout can be continued with the opened mucous fistula: we are used to place a drainable 1-piece drainable transparent pouch (Sensura^®^, Coloplast A/S, Humlebæk, Denmark) connected with a drain bag.

## Results

Thirty-five patients underwent SC for UC between 1987 and 2012. Fifteen patients received a primary mucous fistula; the skin was closed over the closed stump in the remainder 20. Seven patients out of these 20 experienced early stump-related septic complication. We used our approach in each of these patients. All patients were on antibiotic treatment with 500 mg metronidazole iv tid and 1 g cefotaxime iv tid. In five cases, we were able to avoid opening of the rectal stump, and a second stoma was unnecessary. In all of them, we observed a dramatic response within a few hours (mean 6, range 3-13 hours), both in fever and in WBC. Intermittent washout was suspended uneventfully after a mean of 36 (range 24-48) hours. There was no need for opening the tip of the rectal stump in the follow-up and they all underwent successfully pouch surgery after a mean of 7.3 (range 6-8) months. One of the two patients who needed opening of the rectal stump was at risk of developing pelvic septic complication due to marked fragility of the rectum and to poor health status; the other one had persistent fever and high WBC after 12 hours. After opening the closed stump, a prompt improving of symptoms was observed in both. In one of these two patients rectal washout wash carried out after placing a drainable bag on the opened stump, and it succeeded in avoiding a permanent mucous fistula.

## Discussion

SC is the mainstay procedure for complicated and fragile UC patients. It is wise to leave a long rectal/rectosigmoidal stump and to place it subcutaneously in left iliac fossa or at the end of the median laparotomy incision[1][3]: it will be easier to find at subsequent pouch surgery; in case of dehiscence, pelvic septic complications will be avoided by simply opening the tip of the stump, creating a mucous fistula. However, patients undergoing colectomy in emergency settings are often prostrated and a second stoma is perceived as a non-irrelevant nuisance [4]; moreover it could be associated with worst cosmetic results.

Although controversial, the use of intraoperative PVI rectal washout is a widely performed procedure in rectal cancer surgery, and studies reported the efficacy of careful mechanical lavage of the rectal stump in eradicating intraluminal cancer cells shed [5,6]. Some Authors reported the utility of PVI in reducing the bacterial concentration in colon mucosa [7]. A recent review from the Mayo Clinic was sceptical about PVI usefulness in colon surgery, nevertheless the authors concluded that in the acute setting peritoneal or wound irrigation with PVI may be useful in dirty or contaminated operations [8]. It has been suggested that Sodium Hypochlorite is more active on E. coli than PVI [9], but its use should be avoided as it is associated with higher rate of complications [7-10]. However, also colonic irrigation with PVI has some local and systemic effects, as from an animal model: 30 minutes exposure to 5 percent PVI caused severe colonic mucosal injury, with detachment of the epithelial cell layer. Additionally, with the same exposure, 3 litres of 5% PVI whole colonic irrigation showed epithelial desquamation in 11 of 12 patients, which completely healed after three to seven days. No symptoms of acute iodine toxicity were observed, but there was markedly increased urinary iodine and decreased serum thyroid hormones [11,12].

## Conclusion

In our experience, PVI alone was well tolerated and neither systemic nor local side-effects were observed. It was effective in avoiding a second stoma in five out of seven patients, with better quality of life and body perception after IPAA surgery. In one patient with a mucous fistula washout allowed avoiding the formation of a long-term draining stoma. Geriatric patients need to recover completely their physical as well as their mental status before undergoing completion proctectomy with IPAA. Avoiding a second stoma contributed to achieve the aim.

Anyway, we find it prudent to proscribe PVI if there is an history of allergic reaction to iodine-containing compounds, thyroid diseases, renal insufficiency, and established systemic sepsis[8].

## List of abbreviations used

IPAA: ileopouch-anal anastomosis; UC: ulcerative colitis; SC: subtotal colectomy; WBC: white blood cells; PVI: Povidone-Iodine solution.

## Competing interests

Authors have no competing interests to disclose.

## Authors' contributions

GP designed the study, and wrote the draft of the manuscript. GS and GC collected data and participated in the design of the study. SC and FS conceived of the study, and participated in its design and coordination and helped to draft the manuscript. All authors read and approved the final manuscript.
